# Crashworthiness of 3D Lattice Topologies under Dynamic Loading: A Comprehensive Study

**DOI:** 10.3390/ma17071597

**Published:** 2024-03-31

**Authors:** Autumn R. Bernard, Mostafa S. A. ElSayed

**Affiliations:** Mechanical and Aerospace Engineering, Carleton University, Ottawa, ON K1S 5B6, Canada; autumnbernard@cmail.carleton.ca

**Keywords:** energy absorption, finite element analysis, dynamic compression, 316L stainless steel, truss lattice materials

## Abstract

Periodic truss-based lattice materials, a particular subset of cellular solids that generally have superior specific properties as compared to monolithic materials, offer regularity and predictability that irregular foams do not. Significant advancements in alternative technologies—such as additive manufacturing—have allowed for the fabrication of these uniquely complex materials, thus boosting their research and development within industries and scientific communities. However, there have been limitations in the comparison of results for these materials between different studies reported in the literature due to differences in analysis approaches, parent materials, and boundary and initial conditions considered. Further hindering the comparison ability was that the literature generally only focused on one or a select few topologies. With a particular focus on the crashworthiness of lattice topologies, this paper presents a comprehensive study of the impact performance of 24 topologies under dynamic impact loading. Using steel alloy parent material (manufactured using Selective Laser Melting), a numerical study of the impact performance was conducted with 16 different impact energy–speed pairs. It was possible to observe the overarching trends in crashworthiness parameters, including plateau stress, densification strain, impact efficiency, and absorbed energy for a wide range of 3D lattice topologies at three relative densities. While there was no observed distinct division between the results of bending and stretching topologies, the presence of struts aligned in the impact direction did have a significant effect on the energy absorption efficiency of the lattice; topologies with struts aligned in that direction had lower efficiencies as compared to topologies without.

## 1. Introduction

Cellular solids are materials that can be found directly in nature, such as wood or coral, but have also been developed and manufactured in the industrial capacity for a wide range of applications in biological and medical sciences, aviation and aerospace, and defense and automotive industries [1,2,3,4,5,6,7,8]. They are useful for such a variety of applications due to their highly customizable nature; it is possible to tailor one or multiple attributes to obtain a unique and desired set of properties, including high specific stiffness and strength and high energy absorption [1,9,10]. The customization capability of these materials means their tailored set of properties may not be achievable by other existing monolithic materials nor a fully dense solid of the same parent material, and, as such, cellular solids extend the material property selection design space into areas once inaccessible [1,3,11].

Cellular solids are generally defined as solid materials made of cells—which are themselves an assembly of connected struts or plates—which are tessellated in a random (stochastic) or periodic manner to fill a design space [2]. The subset of cellular solids, where the cells are made up of an assembly of plate-like faces and arranged in a stochastic fashion, are generally called *foams* [12]. Conversely, the result of the periodic tessellation of cells is generally called a *lattice material* [3,13,14,15]. This subset of cellular solids—*lattice materials*—are of interest as they typically exhibit superior properties when compared to stochastic cellular solids and, due to their periodic nature, are more controllable and offer greater repeatability and predictability with regards to overall properties and performance [12,13,16,17,18]. Indeed, the advancement in additive manufacturing technology allows for the ability to investigate and characterize these complex geometric materials and for the repeated fabrication of samples and parts, with key features that can be on the micro- or nano-scale [8,15,19,20,21,22,23].

As mentioned, lattice materials are known for their high energy absorption capabilities, which allow them to be excellent candidates for applications such as protective packaging, shock absorption, and crash and blast mitigation, particularly within the aviation and aerospace industries [2,10,12,24,25]. In the literature, typical methods of approach for characterizing and quantifying the behavior of lattice materials—whether subjected to quasi-static or dynamic loading—involve utilizing numerical models, generally developed from or validated by experimental data and/or experimental testing with lattices produced using additive manufacturing.

Nasrullah et al. [26] investigated the dynamic response of eleven topologies (cube, cube open-cell, Kagome, octahedron, octet, pyramid, rhombicuboctahedron, rhombic-dodecahedron, tetrahedron, truncated pyramid, and a twisted octet) modeled for LS-DYNA and designed with material data based on AlSi-12 manufactured using Selective Laser Melting (SLM). Their numerical model results reveal that the topology-optimized octet topology—the “twisted octet”—had the highest specific energy absorption when the deformation mode was bending-dominated (at a relative density of less than 20%) versus when it is stretching-dominated (at a relative density above 25%). This twisted octet topology was then utilized in an aircraft subfloor structure and showed better specific energy absorption than the original design, indicating that the new configuration could be utilized in this type of system.

Mueller et al. [10] utilized ABAQUS/Explicit to characterize the impact performance of four aluminum alloy-based topologies (cube, Delauney, octet, and Voronoi) undergoing quasi-static and dynamic ($<10^{4}/s$) strain rates. They concluded that while relative density was the most important parameter for determining the response of stochastic foams, the deformation mode (bending or stretching) was the most important for periodic structures. By investigating the effect of unit cell rotation with respect to impact direction, they also concluded that this orientation was important for energy absorption properties since the same topology at different orientations might exhibit different deformation modes.

Wang et al. [27] used theoretical and numerical methods to characterize the deformation mode and energy absorption potential of an aluminum alloy FCC lattice at different orientations and impact velocities. The theoretical analysis calculated plateau stresses for the unit cell at different orientations and validated the numerical model, which used the solver ABAQUS. They found that unit cell orientation and impact velocity had an effect on energy absorption, plateau stress, densification strain, and deformation mode. At high impact velocities, however, the deformation mode “I”—defined by the concentration of the deformation in a band perpendicular to the load direction—prevailed regardless of unit cell orientation. For the FCC lattice, an orientation of 45° resulted in the highest energy absorption and plateau stress but the lowest densification strain (and vice versa for a 20° orientation).

Ozdemir et al. in [24,28] observed the energy absorption properties of three titanium alloy-based (Ti6Al4V) topologies (cubic, diamond, and re-entrant cube) under quasi-static and dynamic loading, manufactured using the additive manufacturing technique Electron Beam Melting. First, they performed quasi-static experiments under compression using a universal test machine, while the dynamic tests were performed using a Hopkinson pressure bar. Then, in [28], they created a numerical non-linear finite element model based on the experimental results of the previous paper, finding good accuracy between experimental and numerical results. It is believed that those were the first papers to investigate the dynamic response of the diamond and re-entrant topologies.

Jin et al. [29] additively manufactured samples of four different topologies (two novel diamond lattices,—Dfcc and Dhex—FCC, and BCC) made of titanium alloy Ti6Al4V manufactured with SLM. The dynamic response was tested on a split Hopkinson pressure bar, and the numerical model, created in LS-DYNA, showed a good correlation with the experimental results. They found that the Dfcc and Dhex lattices showed a mixture of stretching- and bending-dominated deformation modes, while the FCC and BCC topologies were stretching- and bending-dominated, respectively. Under dynamic loading, they found that the lattices exhibiting a stretching-dominated deformation mode had better mechanical properties.

Lei et al. [30] fabricated aluminum alloy multi-layer BCC and BCC-Z lattice samples using SLM and, prior to experimental testing, examined the specimens using X-ray micro-computed tomography (μ-CT). This data allowed the novel finite element model they developed to factor in printing imperfections, which are not generally a consideration. Using such a design approach for the lattice within the numerical model, they found that predicted compressive modulus and initial crushing strength were consistent with experimental results.

It is obvious that the design of lattice materials requires the consideration of many multiscale variables, including parent material, geometry and topology, and relative density, among others [1,31]. However, the literature identified previously is generally limited in the variety of topologies examined, a limitation which was also described by Helou and Kara in [3] after a review of over 45 publications in the realm of lattice structures. Helou and Kara also critiqued the difficulty in comparing data from one paper to the next due to the variations in materials and investigative approaches and having no standardized test methods for analyzing lattice structures. Indeed, there are many modeling strategies available for the investigation of lattice structures; Giorgio et al. [32] defined the mechanical behavior of pantographic lattices using a second-grade elasticity model, while Tran and Niiranen [33] and Dong et al. [34] formulated a non-linear Euler–Bernoulli beam model that maintained high accuracy while saving computational costs and developed a numerical homogenization method for 3D cellular materials in MATLAB, respectively.

To begin to close the gap caused by differences in methods between publications, further exacerbated by limited topologies being investigated per publication, this paper analyzes over 20 different topologies using the same base FEA model. To quantify impact performance, we look specifically at energy absorption capabilities—calculating energy absorption efficiency and densification strain—across multiple impact strain rate levels. This work was accomplished by validating the developed FEA model with experimental data and utilizing FEA model material data published and validated in [16,17].

This paper is organized into three main sections following this introduction. In Section 2, the lattice topology design, finite element model, design of experiment approach, and validation are described. In Section 3, the results collected are presented and discussed. Finally, the conclusion is in Section 4, with acknowledgments and references following.

## 2. Materials and Methods

### 2.1. Lattice Geometry and Design

For this study, 24 topologies were investigated. Each topology was modeled as a single unit cell with an original height of 10 mm, where the radius of the struts was varied to investigate three different relative densities, namely 0.10, 0.20, and 0.30. While it is theoretically possible to utilize Equation (1) from [26] to determine the design radius (R) for a given topology based on unit cell height (h) and relative density ($\bar{\rho }$), since k and c are correction coefficients dependent on the topology (k—correction of total strut length of a unit cell, c—correction of total strut length of geometrical cubic), in practice, it is much more difficult [26].

(1) $$\bar{\rho }=k{\left(\frac{R}{h}\right)}^{2}-c{\left(\frac{R}{h}\right)}^{3}$$

To determine those coefficients, one can derive them by observing the geometry of a single strut within the given topology as per the method in [35,36]. For simpler topologies, this derivation is straightforward. Additionally, for certain topologies, such as octet, the procedure and results have been well-documented in the literature [11,31,36,37,38]. However, the same data for other topologies is quite limited or non-existent: [36,38] for octet; [36] for truncated octahedron; [39] for BCC-Z; [39,40] for rhombic dodecahedron; and [41,42] for BCC. Additionally, sources may use a modified version of Equation (1), where instead of the height of the cell, the radius (or even diameter) is normalized against the length of one strut. The differences between the literature and a lack of values for the topologies of interest in this study led to determining the design radius for each of the 24 topologies using a combination of what was already identified in the literature and the homogenization code written in MATLAB from [34]. While the main purpose of that code is to determine the homogenized constitutive matrix of 3D cellular materials, it also outputs a relative density calculated using topology, cell size, and radius during the calculation process. This value was used to determine an approximate radius for geometry creation, which was ultimately confirmed after creating the geometry in ANSYS SpaceClaim (2020 R2) using a modified version of the code from [43].

**Figure 1:**
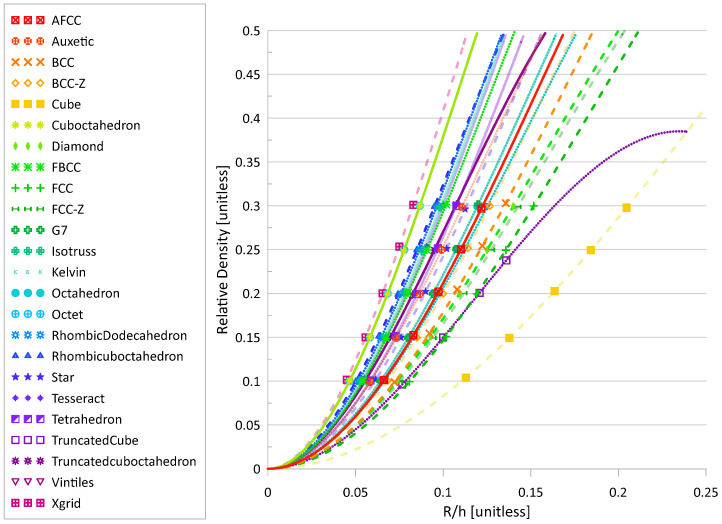
Relative density versus the ratio of radius to unit cell height. Line types distinguish between stretching (solid), bending (dotted), and mixed (dashed) deformation modes, discussed in Section 3.1. Line opacity indicates whether there is at least one strut aligned in the loading direction: opaque—no, semi-translucent—yes.

Radius values and the corresponding relative density for each topology are provided in Table 1, as well as a visual representation of each topology. This data was utilized to compute a curve fit and the coefficients k and c from Equation (1) for the 24 studied topologies, making it easier to determine the radius for a given relative density (or vice versa) in the future. The plot of relative density vs radius–height ratio and the coefficients are provided in Figure 1 and Table 2. The plot is similar to the one presented in [14], though it expands on the number of topologies presented and provides numerical values for the coefficients for use in Equation (1).

### 2.2. Finite Element Model

#### 2.2.1. Initial Model Creation

**Figure 2:**
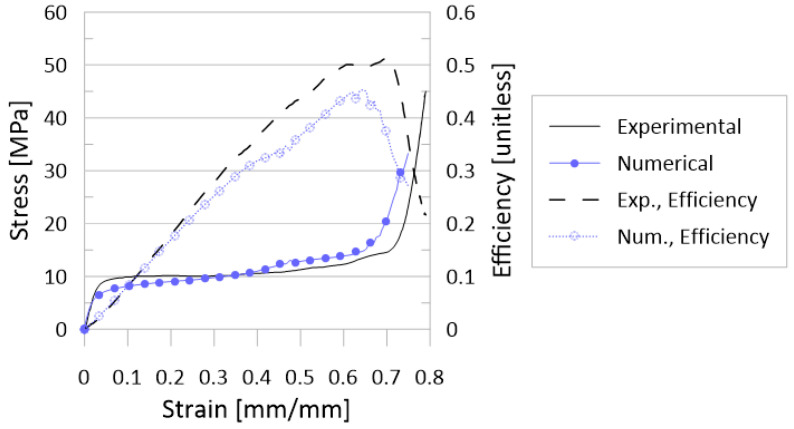
Stress–strain and efficiency–strain results for the quasi-static experiments (from Cao et al. [17]) and the corresponding numerical model as designed for this work. Select deformation behavior illustrated in Figure 3.

**Figure 3:**
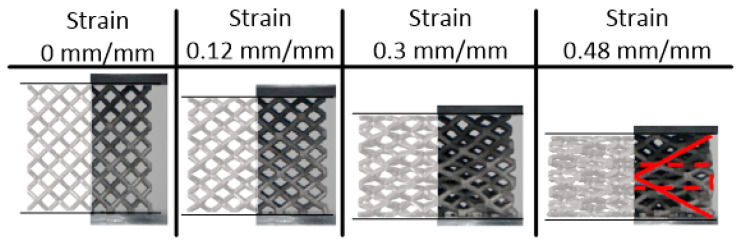
Deformation behavior of rhombic dodecahedron cluster from experiments (half-image on the right, from Cao et al. [17]) and corresponding numerical model for this work (half-images on the left) for given strain values. Corresponding stress and efficiency results are provided in Figure 2.

The finite element model was developed in Altair’s HyperMesh (v2020, Altair Engineering Inc., Troy, MI, USA) for the explicit finite element solver Radioss (v2020, Altair Engineering Inc., Troy, MI, USA). The initial finite element model was based on that model from the literature containing the material model (further described in Section 2.2.4.), which provided quasi-static results for a 3 × 3 ×3 rhombic dodecahedron lattice with overall dimensions of 24 mm × 24 mm × 24 mm and a relative density of 11.68% [17]. As in [40], a one-quarter model was created, reducing the complexity and computation time for the model. Boundary conditions for the one-quarter model along the planes of symmetry were additionally applied following [40]. Figure 2 and Figure 3 illustrate the stress–strain and deformation results, respectively, of the quasi-static (0.001/s) experimental results from Cao et al. [17] and the developed numerical model for this work. Note that stress and strain values are for the lattice as a whole; load and displacement data were utilized, along with original model dimensions, to calculate stress and strain values. These figures suggested a good correlation to the experimental results, initially validating the model. Further requirements to reduce the model complexity (due to the large number of numerical simulations to be completed, detailed in Section 2.3) meant further validation for a model containing a single unit cell, described in the following subsections.

#### 2.2.2. Unit Cell Model

**Figure 4:**
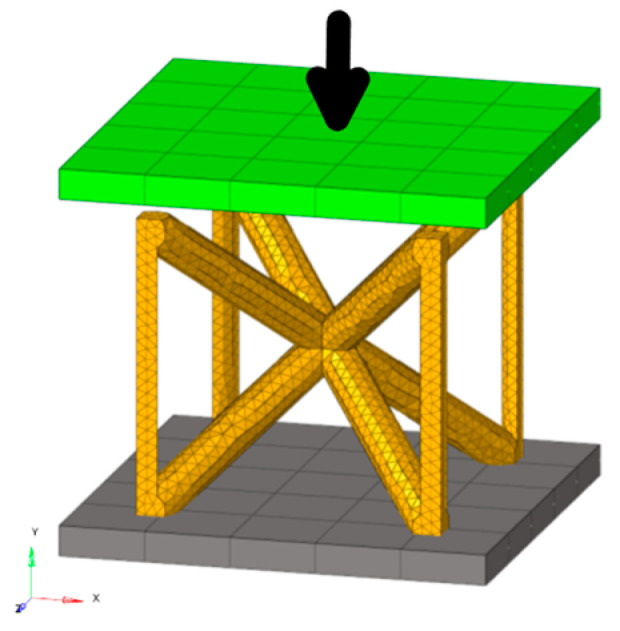
General finite element model components, using a BCC-Z unit cell (orange) for illustrative purposes. Base plate (grey) is fixed and not permitted to translate or rotate. Impactor (green) is given an initial velocity in the downward y-direction as indicated by the black arrow.

The model was constructed such that a single unit cell made of solid tetrahedron elements was impacted at a given initial velocity by a flat plate impactor (“impactor”) made of 4-node shell elements. An additional non-moving, fixed flat plate (“base”) is modeled beneath the single unit cell, illustrated in Figure 4. This model has boundary conditions, as detailed in Section 2.2.3., which represent a single-layer lattice; multi-layer lattice behavior is considered out-of-scope for this work.

Both the impactor and the base have side lengths of 14 mm and thicknesses of 1 mm. They are modeled as rigid, and the mass and initial velocity of the impactor are parametrized, allowing for variations to the initial kinetic energy (“impact energy”). The initial velocity was applied to the primary node of the rigid body, in the middle of the impactor, and only in the direction of axial compression.

For unit cell topologies, it should be noted that while impact only occurs in the global y-direction, for non-isotropic orientations (such as BCC-Z, octahedron, etc.), the results may change depending on the orientation of the unit cell; the investigation of unit cell orientation was beyond the scope of this work.

There are three interfaces defined in the model: (i) the interaction between the impactor and lattice cell, (ii) the self-contact of the lattice cell during compression, and (iii) the interaction between the fixed plate and lattice cell. The former and latter are both controlled by a solid contact interface between a primary surface (impactor or plate) and secondary nodes (lattice cell). The self-contact of the lattice cell was controlled by a single-surface interface.

#### 2.2.3. Boundary Conditions

Considering the Cartesian coordinate system defined in Figure 4, all translational and rotational degrees of freedom of the rigid plate impactor are restricted except the vertical translational motion, as the impactor was only allowed to move along the y-axis. For the lattice unit cell, periodic boundary conditions (BCs) were applied such that they mimicked the periodicity of the lattice. 

**Figure 5:**
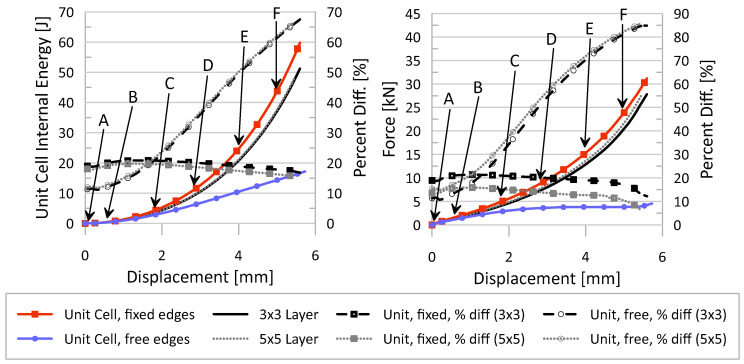
(**Left**) Unit cell internal energy over compression of lattice. (**Right**) Interface force over compression of lattice. Lettered displacement locations (A, B, C, D, E, and F) correspond to images in Figure 6 for the two single-unit cells and the 3 × 3 layer.

**Figure 6:**
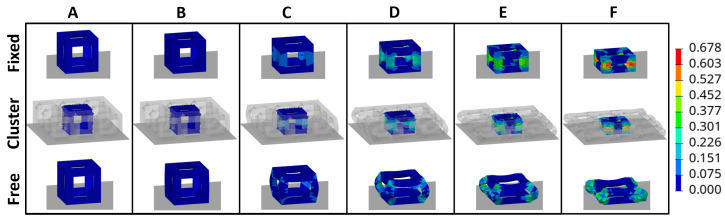
Deformation behavior of two unit cells with different boundary conditions applied to the sides (fixed in top row and free in bottom row), as compared to the middle unit cell of a single-layer 3 × 3 lattice cluster (middle row). Color contour is of plastic strain, units [mm/mm]. Letters correspond to displacement locations in Figure 5.

For validation of these BCs, four separate models were created and tested: a model containing a unit cell whose nodes on the side surfaces were restricted to permit only motion in the y-axis direction; a model containing a unit cell whose nodes on the side surfaces were not restricted; and models containing a single-layer of either 3 × 3 or 5 × 5 clustered unit cells lattice whose nodes on the side surfaces were not restricted. The interest in these clustered lattices was for the comparison of internal energy, force, and general deformation behavior of the middle unit cell to those resulting from the single unit cell models. These results are depicted in Figure 5 for internal energy and force over the course of the compression event and Figure 6 for general deformation behavior characteristics. It should be noted that all models were all compressed at the same rate and the lattices were all defined by the same material parameters presented in Section 2.2.4.

From Figure 5, it can be seen that fixed BCs applied to a single unit cell represent the response of the middle unit cell in a clustered (3 × 3 or 5 × 5) layer most accurately, as compared to the results of the unit cell with free BCs. In general, across the compression event, the percent difference between fixed BC results and the middle unit cell of the cluster is below 20% (absolute value), whereas the free BC model reaches a percent difference of almost 70% and 90% for internal energy and force, respectively. Even the deformation behavior, illustrated in Figure 6, is best captured by the fixed BCs as opposed to the free BCs; the vertical struts of the cubic unit cell in the fixed BC model “squish” in the same manner as those same struts of the middle unit cell in the 3 × 3 cluster while the vertical struts of the unit cell in the free BC model instead buckles outwards.

Such validation testing was also used to justify the use of a single unit cell as opposed to a cluster of unit cells, reducing the overall computation time and memory requirements that would come with the larger lattice model. In Table 3, the number of elements in the lattice, the output file size (animation file and results file), and the overall run time (all simulations were run using parallelization across 32 cores) are provided for the three simulation runs. Being able to utilize one unit cell with the appropriate BCs allows for a minimum 70% to 89% reduction of those measures. As such, all topologies were modeled as a single lattice unit cell for the remainder of this work.

#### 2.2.4. Material and Failure Model

The lattice unit cell was modeled as a 316L stainless steel alloy with properties as produced by the additive manufacturing technique SLM, and, in Radioss, the material itself was defined using a Johnson–Cook strength model [44,45].

The Johnson–Cook strength model considers the plastic stress to be made up of the product of three terms, each considering a different mechanism, namely: strain hardening; strain rate hardening; and temperature softening. Equation (2) presents this product, where A, B, C, m, and n are all material constants, determined from experimental testing, and ${\bar{\varepsilon }}_{p}$, ${\dot{\varepsilon }}^{*}$, and $T^{*}$ are the equivalent plastic strain, the normalized equivalent plastic strain rate, and the dimensionless homologous temperature, respectively. Equations (3) and (4) define the normalized equivalent plastic strain rate and the dimensionless homologous temperature, respectively, where $\dot{\varepsilon }$ is the plastic strain rate, ${\dot{\varepsilon }}_{0}$ is the reference strain rate, $T_{0}$ is room temperature, and $T_{m}$ is melting temperature.

(2) $$\bar{\sigma }=\left[A+B{\bar{\varepsilon }}_{p}^{n}\right]\left[1+C\ln{\dot{\varepsilon }}^{*}\right]\left[1-T^{*m}\right]$$

(3) $${\dot{\varepsilon }}^{*}=\frac{\dot{\varepsilon }}{{\dot{\varepsilon }}_{0}}$$

(4) $$T^{*}=\frac{T-T_{0}}{T_{m}-T_{0}}$$

For this model, no temperature effects were considered. All other material constants required to define the Johnson–Cook strength model for the 316L stainless steel, as manufactured by SLM, are provided in Table 4, including material density ($\rho_{0}$), Young’s modulus (E), and Poisson’s ratio (ν). These values come from the work of Cao et al. in [16,17], where a numerical model employed the Johnson–Cook strength and failure models for the lattice structures and was validated by experimental testing.

As in [16,17], the material card definition for this FEA model also utilized the Johnson-Cook failure model. This failure model splits the plastic portion of the stress–strain behavior into *damage initiation* and *damage evolution* up until the complete failure of the material. Damage initiation can be described by Equation (5), specifically that when ω=1, damage is initiated (ω can vary between 0 and 1).

(5) ω=∑∆ε¯pε¯f

In this equation, $∆{\bar{\varepsilon }}_{p}$ is the increment of the equivalent plastic strain and ${\bar{\varepsilon }}_{f}$ is the equivalent plastic strain at failure. The equivalent plastic strain at failure can be defined by Equation (6), where $D_{1}$ through $D_{5}$ are material constants and $\sigma^{*}$ is the ratio of the mean stress ($\sigma_{mean}$) to the Mises equivalent stress ($\sigma_{eff}$). The material constants $D_{1}$ through $D_{5}$ are presented in Table 4. As with the constants for the Johnson–Cook strength model, these values come from the work of Cao et al. in [16,17], and temperature effects are not considered.

(6) $${\bar{\varepsilon }}_{f}=\left[D_{1}+D_{2}\exp\left(D_{3}\sigma^{*}\right)\right]\left[1+D_{4}\ln{\dot{\varepsilon }}^{*}\right]\left[1+D_{5}T^{*}\right]$$

### 2.3. Design of Experiments

The energy absorption capabilities of each lattice topology unit cell and relative density were observed through four variations to impactor initial kinetic energy (KE) and four variations to impactor initial velocity for a total of 16 different initial velocity-KE combinations per a given topology and relative density set. An “energy matrix” is presented in Table 5, which outlines the different speed and impactor energy values (and corresponding impactor mass) for each of the 16 different runs. The 16 Radioss files per topology and relative density pair were created from the “parent” HyperMesh models by utilizing commands in .tcl files to modify the mass and velocity parameters of the impactor. Once passed through the solver, the animation and time history files were processed, and result data was collected using a developed .oml file in Altair’s Compose (v2021, Altair Engineering Inc., Troy, MI, USA). The final post-processing of the collected data was performed in MATLAB (R2020b, MathWorks, Natick, MA, USA) and Golden Software’s Grapher (20.1.251, Golden Software LLC, Golden, CO, USA).

## 3. Results

### 3.1. Data Analysis and Crashworthiness Parameters

Data was collected from the finite element solver so that an analysis of the crashworthiness of the lattices could be performed. The determination of the performance of those lattices involved calculating the following crashworthiness parameters:

**Energy Absorption Efficiency.** Also, “EA efficiency” or “efficiency”, this parameter, η, is calculated from the stress–strain (σ–ε) curve as

(7) $$\eta \left(\varepsilon \right)=\frac{1}{\sigma (\varepsilon )}\int_{0}^{\varepsilon }\sigma \left(\varepsilon \right)d\varepsilon$$

where, in the numerical model, the stress is obtained from the impact force between the impactor and lattice divided by the surface area of the upper surface of the lattice unit cell envelope, and the strain is obtained from the displacement of the uppermost surface of the lattice divided by the lattice unit cell height, a method commonly used in the literature [25,46,47].

**Densification Strain.** Using the energy absorption efficiency method, the densification strain, $\varepsilon_{D}$, is

(8) $${\left.\frac{d\eta (\varepsilon )}{d\varepsilon }\right|}_{\varepsilon =\varepsilon_{D}}=0$$

which is simply the strain at the maximum efficiency point ($\eta_{max}$) [25,48,49].

**Plateau Stress.** Plateau stress, $\sigma_{pl}$, along with densification strain, are considered to be the most important values with regard to the energy absorption of materials [50]. This stress is determined using [25]

(9) $$\sigma_{pl}=\frac{\int_{0}^{\varepsilon_{D}}\sigma \left(\varepsilon \right)d\varepsilon }{\varepsilon_{D}}$$

**Energy Absorption.** Energy absorption, *EA* or *IE* (internal energy), is calculated as

(10) $$EA=V\int_{0}^{\varepsilon_{D}}\sigma \left(\varepsilon \right)d\varepsilon$$

It should be noted that while Equation (10) utilizes the densification strain in the integral for calculation, the actual selected strain values do vary in the literature [10,25,26,31,51].

**Specific Energy Absorption.** Specific Energy Absorption (*SEA*) is calculated either per unit volume—${SEA}_{V}$—or per unit mass—${SEA}_{m}$. Equations for both of these specific energy absorptions are provided as follows [25,51,52]

(11) $${SEA}_{V}=\frac{EA}{V}=\int_{0}^{\varepsilon_{D}}\sigma \left(\varepsilon \right)d\varepsilon$$

(12) $${SEA}_{m}=\frac{EA}{m}=\frac{\int_{0}^{\varepsilon_{D}}\sigma \left(\varepsilon \right)d\varepsilon }{\bar{\rho }\rho_{0}}$$

where V is the volume of the lattice structure, and m is the mass of the lattice structure. It should be noted that these values are also calculated up to the densification strain.

**Bending- versus Stretching-Dominated Behavior.** Maxwell’s stability criterion [53] can be utilized to help understand the deformation behavior of a topology, which can be helpful in understanding appropriate applications for a lattice made of that topology. However, it should be noted that even if the stability criterion designates the unit cell of a certain topology as bending-dominated, it is still possible that the actual behavior of the topology is stretching-dominated (or mixed-mode), particularly since the criterion does not account for the loading direction, including the presence of struts aligned directly in the loading direction [54,55]. Indeed, Calladine [56] described *tensegrity* structures as “[constituting] a paradoxical exception to Maxwell’s rule,” going on to examine the conditions required to “break” Maxwell’s rule. In 1986, Peregrino and Calladine [57] discussed a modified version of this criterion, and it has been noted the criterion is only a necessary condition, and not a sufficient condition, for determining truss stiffness [1,58]. However, the determination of the variables for the modified criterion is not trivial [57] and is considered to be beyond the scope of this work. Instead, with this knowledge of the criterion and its limitations, the determination of the compression behavior of the topologies in this work considers several characteristics: Maxwell’s criterion for a single unit cell, strut orientation with respect to loading, and classification in the literature. A summary of these quantitative and qualitative characteristics is provided in Table 6. As can be seen from Table 6, Maxwell’s stability criterion may not accurately reflect the compressive behavior of a topology if it is predicted to be bending-dominated by the criterion. However, those topologies that are determined to be stretching-dominated by the criterion will still exhibit predominantly stretching-dominated behavior during compression.

### 3.2. Numerical Model Results and Discussion

All performance parameters mentioned in the previous section, as well as time-history internal and kinetic energies, force, and displacement data, were carefully analyzed for all 24 lattice topology unit cells at three relative densities for each of the 16 speed-impact energy scenarios of the Design of Experiments. There are four main independent variables in this study, namely, (i) impact KE, (ii) impact speed, (iii) relative density, and (iv) topology—the effects of which are further discussed in the following subsections.

#### 3.2.1. Effect of Initial KE

**Figure 7:**
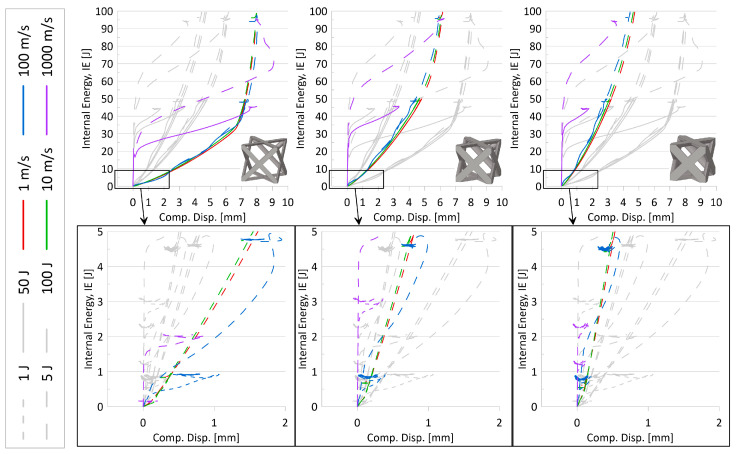
Internal energy over compression displacement for AFCC topology at three relative densities, four speeds, and four initial kinetic energies. Top graphs are for initial KE of 50 J and 100 J, and bottom graphs are for initial KE of 1 J and 5 J (identified by line type per legend). Variations in initial impact speeds are distinguished using the line color specified in legend. From left to right: relative density 10%, 20%, 30%; images of unit cell provided for reference. Grey lines are used to help compare relative densities.

**Figure 8:**
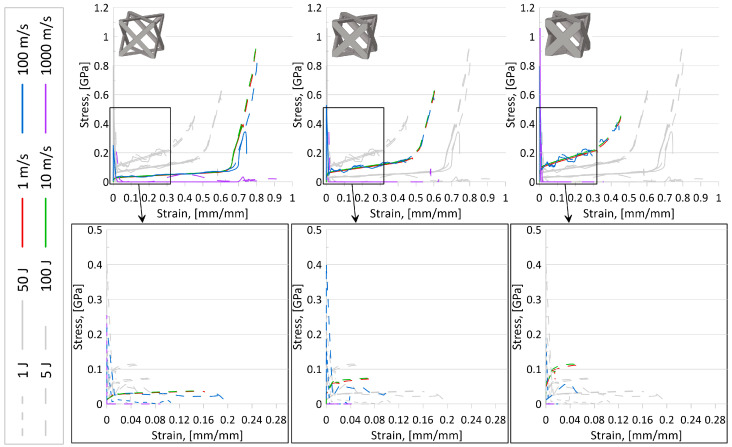
Stress over strain for AFCC topology at three relative densities, four speeds, and four initial kinetic energies. Top graphs are for initial KE of 50 J and 100 J, and bottom graphs are for initial KE of 1 J and 5 J (identified by line type per legend). Variations in initial impact speeds are distinguished using the line color specified in legend. From left to right: relative density 10%, 20%, 30%; images of unit cell provided for reference. Grey lines are used to help compare relative densities.

Based on internal energy variations over the compression event and stress–strain results for all topologies, the following observations were made:

- It was noted that simulations with lower initial impactor kinetic energies mimicked the initial behavior of the larger initial KE, particularly for simulations with initial impactor speeds of 100 m/s or less. That is, the results suggest that running simulations at a larger initial KE could still predict the behavior of the simulations at a lower initial KE. A set of internal energy over compression displacement curves for the AFCC topology (at all three relative densities) is provided in Figure 7 to illustrate such a phenomenon. In the top graphs of this figure, it is evidently seen that the lower initial impact energy curves (solid lines) follow the path of the higher initial internal energy curves (dashed lines) up until the system limit is reached and the higher energy curves continue.
- In looking at stress–strain results, it was revealed that initial impactor KE of 1 J and 5 J was not sufficiently large to reveal complete elastic-plateau-densification stress–strain curves and a demonstratory set of stress–strain curves for the AFCC topology is provided in Figure 8. As a result, calculated crashworthiness parameters—such as densification strain or energy absorption—for the simulations with those lower initial KE would not accurately reflect the behavior and capabilities of the topology. It should be noted that for some topologies, even an initial impactor KE of 50 J was not always sufficient to reveal the complete elastic-plateau-densification characteristics expected of a stress–strain curve for lattice materials, particularly at higher relative densities.

Based on such observations—which suggest that the full potential of the topology is not being utilized if the impactor initial KE is lower and that the curves for low KE mimicked the behavior of the larger KE anyway—the focus for further investigations was narrowed to include only results from the 100 J initial KE simulations.

#### 3.2.2. Effect of Speed

**Figure 9:**
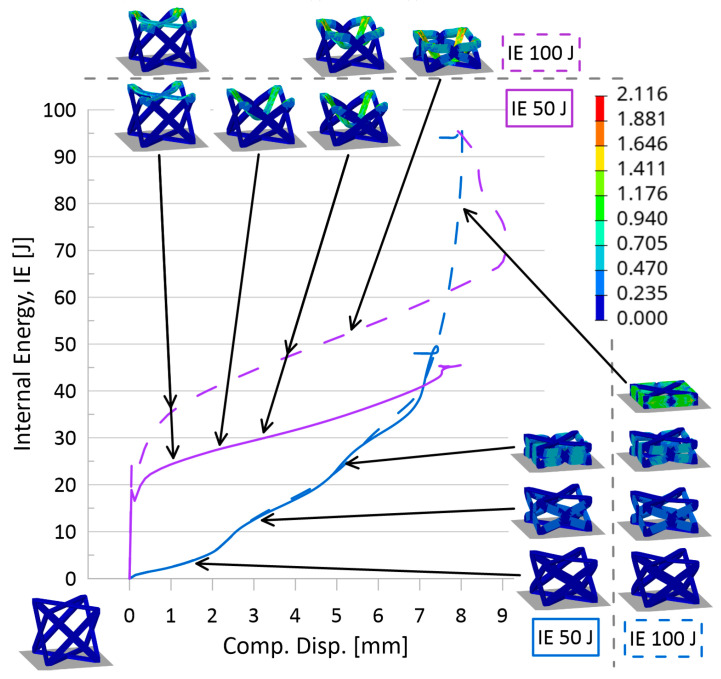
Internal energy over compression displacement for AFCC topology at a relative density of 10%. Variations in initial KE are 50 J and 100 J (distinguished by line type). Variations in initial impact speeds are 100 m/s and 1000 m/s (distinguished by line color). Color contour for images of compression of unit cells is for plastic strain [mm/mm].

Based on internal energy variations over the compression event and stress–strain results for all topologies, the following observations were made:

- As illustrated in Figure 7, despite the strain-sensitivity of the material, up to initial impactor speeds of approximately 10 m/s, there is very little difference in results, suggesting that at those low speeds, the sensitivity of the material to strain rate variations is negligible. While it also appears that data from simulations with initial impactor speeds of 100 m/s is also insignificantly different from the two lower speed levels in those plots, stress–strain plots for certain topologies (as an example, in Figure 8) did suggest that results from 10 m/s could not always be used to accurately predict results for a speed of 100 m/s.
- Also seen from stress–strain results was the indication that the current model setup was insufficient to appropriately reveal topology behavior at an initial impactor speed of 1000 m/s. While the internal energy over the compression event curves (Figure 7) and associated simulation animations (Figure 9) for numerical models at initial impactor speeds at 1000 m/s appear to illustrate the phenomenon of layer-by-layer collapse at high impact speeds (versus all layers deforming in the same or similar manner during a quasi-static or low-speed compression event), the jump in magnitude from 100 m/s to 1000 m/s results in erratic time-history behavior, bringing into question the reliability and credibility of the data. Additionally, with only one unit cell contained in this model, such a conclusion would realistically require multiple layers. Coupled with the stress–strain result observations, data for numerical models with impact speeds of 1000 m/s were perceived to have a high level of uncertainty.

Based on the observations for the effect of speed and initial KE, the focus for additional data analysis was placed on simulations with an initial KE of 100 J at speeds of 10 m/s and 100 m/s.

#### 3.2.3. Effect of Relative Density

To first get a sense of the static mechanical properties of the topologies of interest in this work, the homogenized properties—such as the constitutive matrix and Young’s modulus (in x-, y-, and z-axes directions)—were computed for all topologies at the three relative densities investigated (10% through 30%, at 10% increments) using the homogenization code for cellular materials in MATLAB presented in Dong et al. [34] and a modified version of the MATLAB code for visualizing elastic anisotropy from Nordmann et al. [69]. In analyzing the dynamic response of these topologies, this homogenized data was useful in understanding their relationships and behavior.

**Figure 10:**
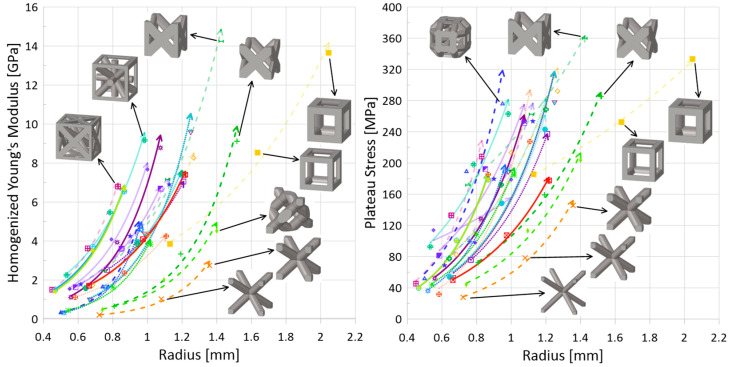
(**Left**) static homogenized Young’s modulus (i.e., in compression direction) versus strut radius and (**right**) plateau stress versus strut radius (impact energy 100 J, speed 10 m/s). Legend as shown in Figure 1. Line types distinguish stretching (solid), bending (dotted), and mixed (dashed) deformation modes, discussed in Section 3.1. Line opacity indicates whether there is at least one strut aligned in the loading direction: opaque—no, semi-translucent—yes (see Table 6 for clear classification on whether strut(s) are aligned in loading direction or not). The arrow at the end of the line indicates increasing relative density.

Plateau stress results have been plotted in Figure 10 alongside the homogenized Young’s modulus results (in the compression direction). It is seen that those topologies, such as cubic (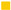) and FCC-Z (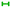), that have higher homogenized strengths for a given relative density also have higher resulting plateau stresses, and understandably, as relative density increases, the plateau stress increases as well. Notably, the rate of increase for the tesseract topology (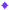) is lower in comparison to other topologies. While the data for the 10 m/s speed and initial impact energy of 100 J is presented in Figure 10, it is noted that, except for actual numerical result values, the trends and relations between topologies are generally the same for a speed of 100 m/s.

**Figure 11:**
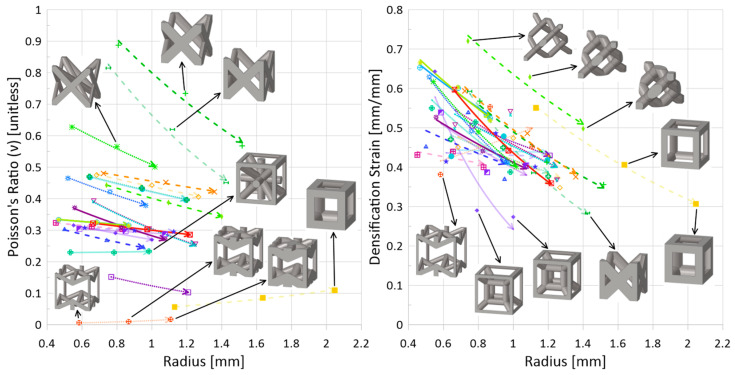
(**Left**) homogenized Poisson’s ratio (in compression direction) versus strut radius and (**right**) densification strain versus strut radius (impact energy 100 J, speed 10 m/s). Legend as shown in Figure 1. Line types distinguish stretching (solid), bending (dotted), and mixed (dashed) deformation modes. Line opacity indicates whether there is at least one strut aligned in the loading direction: opaque—no, semi-translucent—yes (see Table 6 for clear classification on whether strut(s) are aligned in the loading direction or not). The arrow at the end of the line indicates increasing relative density.

**Figure 12:**
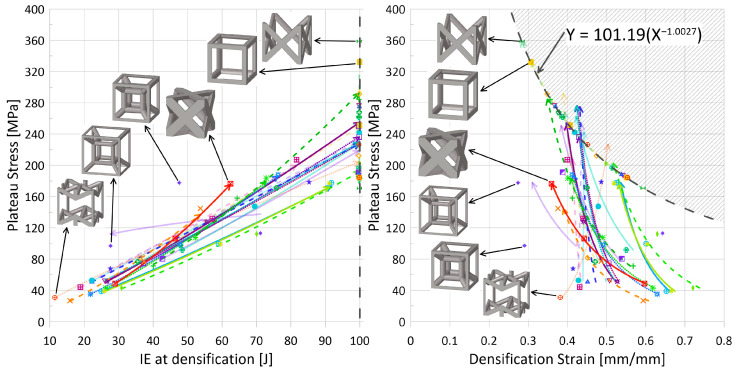
(**Left**) plateau stress versus IE at densification strain and (**right**) plateau stress versus densification strain. Both sets of data are for an impact energy of 100 J, speed of 10 m/s. Legend as shown in Figure 1. Line types distinguish stretching (solid), bending (dotted), and mixed (dashed) deformation modes. Line opacity indicates whether there is at least one strut aligned in the loading direction: opaque—no, semi-translucent—yes (see Table 6 for clear classification on whether strut(s) are aligned in the loading direction or not). The arrow at the end of the line indicates increasing relative density.

**Figure 13:**
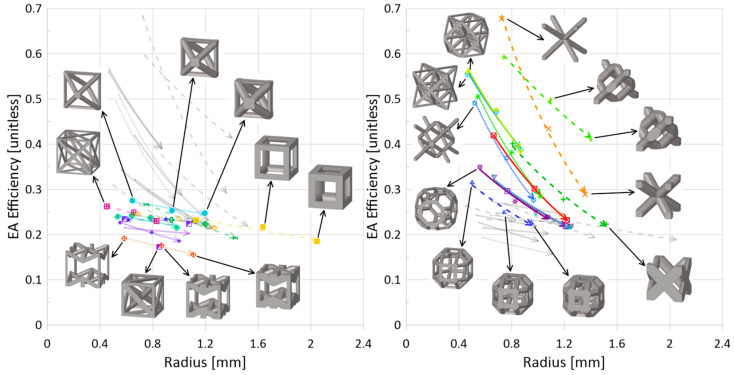
EA efficiency versus strut radius (impact energy 100 J, speed 10 m/s)—left: struts in loading direction; right—no struts in loading direction (see Table 6 for clear classification on whether strut(s) are aligned in loading direction or not). Legend as shown in Figure 1. Line types distinguish stretching (solid), bending (dotted), and mixed (dashed) deformation modes. The arrow at the end of the line indicates increasing relative density. Grey lines are for ease of comparison between the data in the two plots (grey data is found as color data in the other plot).

In addition to an increase in plateau stress for an increase in relative density, the following trends were also generally observed, regardless of topology:

- *Decrease in densification strain*. Shown in Figure 11 (right), this statement is true for all topologies, except auxetic (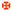). The expectation for this decrease with an increase in relative density is understandable if one considers densification strain to represent the point at which the lattice has been compressed and begins behaving like the solid parent material; at lower relative densities, the lattice must be compressed *more* before it self-contacts and can behave as a monolithic material. Thus, the auxetic behavior is intriguing and will be discussed further in the following subsection.
- *Increase in absorbed energy up to densification strain*. Such a trend can be seen in Figure 12 (left). Since absorbed energy is related to both stress and strain, and it has been observed that the plateau stress increased with increasing relative density, this result is also unsurprising. However, given that the rate at which the plateau stress increased for the tesseract topology was notably lower in comparison to other topologies, the absorbed energy actually *decreased* with an increase in relative density from 0.1 to 0.2; its decrease in densification strain was enough to cause a shift in direction. Additionally, the two highest-strength (and highest plateau-stress) topologies—cubic and FCC-Z—had a consistent internal energy at densification regardless of relative density; had the initial impact energy been increased beyond 100 J, it may have been possible for the trend of *increasing IE for increasing relative density* to be observed.
- *Decrease in energy absorption efficiency*. This decreasing trend can be seen in Figure 13, and more discussion regarding the EA efficiency is provided in the following subsection with regards to whether there are struts aligned in the loading direction.

#### 3.2.4. Effect of Topology

For a mass comparison of the crashworthiness parameters of the 24 topologies, Figure 12 and Figure 13 illustrate some of the interesting relationships observed within the data.

- *Bending versus Stretching*. While bending-dominated cellular materials are known to be better energy absorbers and be compliant (lower strength and stiffness), and stretching-dominated cellular materials have higher stiffness and strength, the dynamic data collected for this work using the numerical model set up described suggests no noticeable delineation between these two categories of topologies (nor mixed-mode topologies), though it is noted that absorbed energy is measured up to the densification of the given topology, not up to a stationary strain point. Additionally, as has been noted by others in the literature, it appears that there is a non-negligible influence of the strut orientation with respect to the loading direction when it comes to deformation modes, which is not accounted for in Maxwell’s criterion and can increase the strength of otherwise bending-dominated topologies.
- *Strut(s) aligned in loading direction*. With regards to energy absorption efficiency (Figure 13), there is, quite interestingly, a clear separation of performance when it comes to a topology having strut(s) aligned in the loading direction versus a topology not having any struts aligned in that direction. For those topologies with struts aligned in the loading direction—
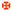

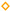

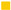

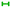

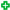

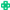

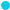

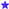

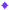

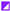

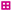
, Figure 13 (left)—the resulting maximum energy absorption efficiency during impact is lower than for topologies that do not have struts aligned in that direction (Figure 13 (right), 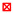

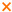

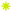

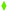

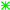

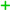

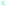

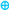

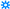

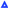

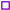

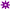

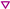
). For plateau stress (Figure 10 (right)), it is obvious that the cubic unit cell—which has struts aligned in the compression direction and fewer struts, leading to a larger overall strut radius to reach a given relative density—has a greater strength than other topologies when considering only a given relative density. Indeed, the topology that has the next highest plateau stress for a given relative density is FCC-Z, which also has struts aligned in the impact direction and has a similarly large radius for struts given the fewer total number of struts compared to other topologies (not including cubic). The BCC (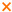) topology, which has no struts aligned in the compression direction and is generally bending-dominated, has the lowest strength.
- *Topology Highlight: Auxetic (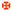).* Unlike other topologies, the densification strain increased with an increase in relative density for the auxetic topology. The Poisson’s ratio of this topology in relation to other topologies—Figure 11 (left)—is notably significantly smaller, almost equal to zero (generally, auxetic materials are those materials with negative Poisson’s ratio, unlike other types of topologies).
- *Topology Highlight: Cubic* (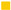) *and FCC-Z* (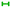). These two topologies had noticeably larger plateau stresses for a given relative density than other topologies. For a constant relative density and unit cell height, these two topologies have the largest strut radius as compared to other topologies with struts aligned in the impact direction. Thus, the combination of large strut radius and struts aligned in the loading direction seems to have allowed for an increase in overall strength and higher plateau stress during impact. Notably, observing the response of the FCC-Z, FCC (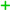), diamond (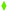), and BCC (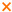) topologies at a constant strut radius of approximately 1.4 mm in Figure 10 (left or right), it is the topology with struts aligned directly in the loading direction (FCC-Z) that has the highest Young’s modulus and plateau stress, whereas the others reach less than 70% of those properties of the FCC-Z topology. Also interesting is that the cubic topology requires a larger strut radius to reach a similar strength and plateau stress as compared to the FCC-Z topology; at a radius of approximately 1.4 mm, it yields similar results to the FCC, diamond, and BCC topologies.
- *Topology Highlight: Tesseract* (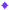). The tesseract topology was noted to have a lower rate of increase in plateau stress for an increase in strut radius (thus, relative density) (Figure 10 (right)). Together with a decrease in densification strain from a relative density of 0.1 to 0.2, the absorbed energy up to densification actually decreased with the increase in relative density (71.2 J vs. 27.6 J). However, with an additional 10% relative density from 0.2 to 0.3, the absorbed energy also increased (to 47.6 J), the results of which are plotted in Figure 12 (left).
- *Topology Highlight: Octahedron* (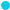)*, Truncated Cube* (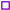)*, Truncatedcuboctahedron* (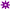)*, Rhombicuboctahedron* (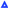). Of the topologies with struts aligned in the impact direction, the octahedron topology is the only one that has two half-struts aligned in that direction, as opposed to four-quarter struts. Interestingly, the energy absorption efficiency results from Figure 13 (left) place the octahedron topology along the *strut-no strut* division. Also close to this split are the truncated cube, truncatedcuboctahedron, and rhombicuboctahedron (Figure 13 (right)), which do not have full struts aligned in the impact direction (i.e., a strut with a length equal to the unit height) but do have smaller-length struts aligned in the impact direction. Such an observation seems to suggest that while it is possible to observe a generally distinct division between the energy absorption efficiency results of Figure 13 based on whether or not there are struts aligned directly in the loading direction, the amount and length of those struts can shift the efficiency.

#### 3.2.5. Discussion Concluding Remarks

Figure 12 plots plateau stress versus both absorbed energy up to densification and the actual densification strain. As expected, Figure 12 (left) shows a positive relationship between increasing plateau stress and absorbed energy—a higher plateau stress means more energy is absorbed during deformation. However, the energy of the system was limited by the initial impact energy of the impactor—100 J for Figure 12 —and, as such, there were some topologies/relative densities that, at densification, had absorbed the impact energy. During analysis of the data, there appears to be a relationship between the impact energy, plateau stress, and densification strain, shown in Figure 12 (right) with the limiting grey dashed curve. For a given initial impact energy, there were densification-plateau stress pairings that were unattainable; for an initial impact energy of 100 J, this area has been highlighted in Figure 12 (right), and the limiting curve equation has been provided. Other limiting curves for the lower initial impact energies—1 J, 5 J, 50 J—were also observed; they had similar power exponents (approximately equal to −1) but lower coefficients (due to their lower initial impact energies).

While this work dealt with results predicted by a developed numerical model, the material data itself came from additively manufactured samples. Since additive manufacturing technologies have greatly expanded the ability to research lattice materials, it is important to note their limitations, particularly in regard to minimum geometrical dimensions, such as the strut radii. From the results for these 24 topologies across the range of relative densities, it can be seen that for the range of strut diameters (approximately 0.8 mm to 4.4 mm), there is a variety of mechanical property sets that could be obtained simply by modifying the topology: Figure 10 (right) indicates that the plateau stress of a cube (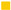) at a relative density of 10% is almost equivalent to the plateau stress of an AFCC topology (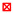) at a relative density of 30%, allowing for a reduction in structure weight; Figure 11 (right) indicates that there is a minimal change in densification strain for changing relative density for the Xgrid topology (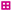); and Figure 13 shows that by simply removing the struts aligned in the impact direction, the energy absorption increases.

## 4. Conclusions

In an attempt to close the gap in the literature caused by the difficulty in comparing data between papers due to material, model, and setup differences, this work analyzed the dynamic impact performance of over 1,000 lattice simulations, which included over 24 topologies at three relative densities. To ensure the data captured represented a wide dynamic range, four different impact speeds and initial impact energy levels (each) were simulated using a developed numerical model containing a single unit cell with a material model sensitive to strain rate and validated boundary conditions. Several conclusions based on careful analysis of time-history-based data as well as crashworthiness parameters, including energy absorption and densification strain, were drawn:

- Despite the strain-rate-sensitive material properties of the SLM steel alloy, changes in impact performance from 1 m/s to 10 m/s were negligible (for the same initial impact energy). There were some changes between speeds of 10 m/s and 100 m/s, but general trends were similar regardless of the data set observed. At speeds of 1000 m/s, significant performance changes were observed, but it was determined that there was a large amount of uncertainty in results given the current numerical model setup—single unit cell with periodic boundary conditions—and that a multi-layered, larger cluster of cells would probably be better suited to illustrate the layer-by-layer collapse observed for high impact speeds with lattice materials.
- Increasing initial impact energy allowed for a better representation of the elastic-plateau-densification stress–strain curve characteristic of lattice materials. Lower impact energies whose stress–strain curves did not reach densification simply revealed a portion of the stress–strain curve; higher energies were better for performance comparison, given more representative values for densification strain and energy absorption up to densification.
- For increasing relative density, it was generally seen that the plateau stress increased, the densification strain decreased, the energy absorbed at densification increased, and the energy absorption efficiency decreased. A few exceptions include the auxetic and tesseract topologies.
- It was clear that topologies with struts aligned in the impact direction had lower energy absorption efficiencies as compared to topologies that had no struts aligned in that direction.
- Based on the numerical model setup, there was no clear separation in performance based on bending, stretching, or mixed deformation modes.

## Figures and Tables

**Table 1:** Geometry and design data for lattice structures.

Topo.	Geometry	$\bar{\rho }$	R [mm]	Topo.	Geometry	$\bar{\rho }$	R [mm]
AFCC	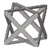	0.1	0.663	Kelvin	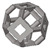	0.1	0.665
		0.2	0.973			0.2	0.985
		0.3	1.218			0.3	1.250
Auxetic	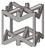	0.1	0.583	Octahedron	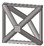	0.1	0.645
		0.2	0.867			0.2	0.945
		0.3	1.108			0.3	1.195
BCC	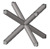	0.1	0.723	Octet	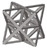	0.1	0.466
		0.2	1.080			0.2	0.684
		0.3	1.357			0.3	0.867
BCC-Z	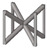	0.1	0.683	Rhombic Dodecahedron	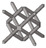	0.1	0.519
		0.2	1.000			0.2	0.760
		0.3	1.267			0.3	0.965
Cube	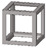	0.1	1.130	Rhombi-cuboctahedron	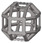	0.1	0.500
		0.2	1.636			0.2	0.742
		0.3	2.047			0.3	0.950
Cuboctahedron	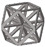	0.1	0.467	Star	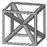	0.1	0.615
		0.2	0.683			0.2	0.900
		0.3	0.867			0.3	1.125
Diamond	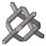	0.1	0.740	Tesseract	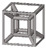	0.1	0.551
		0.2	1.095			0.2	0.792
		0.3	1.400			0.3	1.000
FBCC	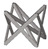	0.1	0.542	Tetrahedron	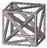	0.1	0.585
		0.2	0.800			0.2	0.850
		0.3	1.017			0.3	1.075
FCC	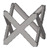	0.1	0.808	Truncated Cube ^1^	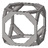	0.1	0.767
		0.2	1.192			0.2	1.208
		0.3	1.517				
FCC-Z	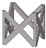	0.1	0.750	Truncatedcuboctahedron	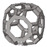	0.1	0.558
		0.2	1.117			0.2	0.825
		0.3	1.425			0.3	1.075
G7	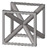	0.1	0.642	Vintiles	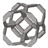	0.1	0.667
		0.2	0.942			0.2	0.983
		0.3	1.200			0.3	1.250
IsoTruss	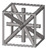	0.1	0.533	Xgrid	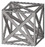	0.1	0.450
		0.2	0.783			0.2	0.655
		0.3	0.983			0.3	0.830

^1^ The truncated cube cannot be built beyond a relative density of ~24%.

**Table 2:** Coefficients k and c for the equation $\bar{\rho }=k{\left(\frac{R}{h}\right)}^{2}-c{\left(\frac{R}{h}\right)}^{3}$, where $\bar{\rho }$ is unitless, R is radius, and h is unit cell height, and both have the same units.

Topology	k	c	Topology	k	c
AFCC	26.656	54.618	Kelvin	26.657	60.331
Auxetic	34.381	90.751	Octahedron	28.049	59.170
BCC	21.765	39.187	Octet	53.313	154.493
BCC-Z	24.907	49.179	Rhombic Dodecahedron	43.530	117.566
Cube	9.425	11.311	Rhombicuboctahedron	46.847	144.491
Cuboctahedron	53.313	154.493	Star	31.190	69.157
Diamond	21.765	46.361	Tesseract	39.156	90.673
FBCC	39.542	102.212	Tetrahedron	33.635	71.375
FCC	17.774	31.282	Truncated Cube	20.919	59.354
FCC-Z	20.913	43.497	Truncatedcuboctahedron	39.391	123.597
G7	28.048	59.169	Vintiles	26.657	60.330
IsoTruss	40.614	99.127	Xgrid	170.547	57.846

**Table 3:** Number of elements in model, output file size, and run time for unit cells and single-layer cluster, along with associated percent reductions.

Model	Num. Elem.	Output File Size [MB]	Run Time [min]	Percent Reduction ^1^ [%]		
				Num. Elem.	Output File Size	Run Time
Free BC	3139	74	15	89	85	72
Fixed BC	3139	74	16	89	84	70
3 × 3 Single-Layer Cluster	27,520	498	53	-	-	-
5 × 5 Single-Layer Cluster	77,547	1010	339	-	-	-

^1^ As compared to the 3 × 3 cluster.

**Table 4:** Mechanical properties of 316L stainless steel alloy (manufactured by SLM) for use in Johnson–Cook strength and failure models.

	$\rho_{0}$	E	ν	
	7960 kg/m^3^	93 GPa	0.3	
A	B	C	n	${\dot{\varepsilon }}_{0}$
310 MPa	622	0.1	0.8	0.001
$D_{1}$	$D_{2}$	$D_{3}$	$D_{4}$	$D_{5}$
0.1152	1.0116	−1.7684	−0.05279	0.5256

**Table 5:** Speed-kinetic energy matrix for the 16 runs per topology-relative density, with mass [kg] in the center.

Strain Rate ^1^ [1/s]	Speed [m/s]	Initial Kinetic Energy [J]			
		1	5	50	100
100	1	2	10	100	200
1000	10	0.02	0.1	1	2
10,000	100	0.0002	0.001	0.01	0.02
100,000	1000	0.000002	0.00001	0.0001	0.0002

^1^ Strain rate is calculated by dividing speed by the height of the unit cell (10 mm).

**Table 6:** Topologies as bending- or stretching-dominated and whether the topology has struts aligned directly in the loading direction.

	Topology		b ^1^	j ^2^	M ^3^	S/B ^4^	S/B ^5^	Ref.	LD? ^6^
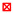	AFCC	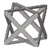	24	12	−6	B	S	[59]	N
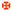	Auxetic	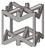	36	18	−12	B	B	[60,61]	Y
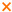	BCC	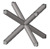	8	9	−13	B	S/B	[14,29,54,55]	N
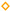	BCC-Z	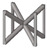	12	9	−9	B	S/B	[54,55]	Y
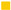	Cube	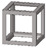	12	8	−6	B	S/B	[10,14,26,62]	Y
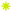	Cuboctahedron	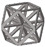	36	13	3	S	S	[60]	N
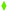	Diamond	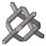	16	14	−20	B	S/B	[14,24,28]	N
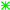	FBCC	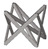	24	13	−9	B	B	[60]	N
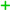	FCC	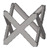	16	12	−14	B	S/B	[54,55]	N
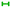	FCC-Z	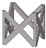	20	12	−10	B	S/B	[54,55]	Y
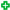	G7	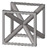	16	9	−5	B	B	[60,63]	Y
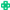	IsoTruss	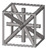	26	15	−13	B	S	[10,59]	Y
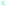	Kelvin	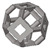	36	24	−30	B	B	[10,14,64,65]	N
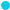	Octahedron	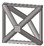	12	6	0	S	S	[26,59]	Y
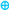	Octet	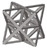	36	14	0	S	S	[10,26,29,59,65]	N
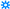	Rhombic Dodecahedron	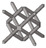	32	20	−22	B	B	[16,40]	N
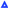	Rhombicuboctahedron	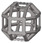	48	24	−18	B	S/B	[14,26,64]	N
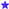	Star	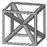	20	9	−1	B	S/B	[14,59]	Y
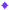	Tesseract	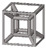	32	16	−10	B	S	[60,66,67]	Y
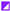	Tetrahedron	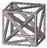	22	9	1	S	S	[68]	Y
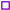	Truncated Cube	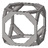	36	24	−30	B	B	[14]	N
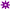	Truncatedcuboctahedron	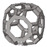	72	48	−66	B	S	[60]	N
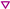	Vintiles	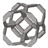	36	28	−42	B	B	[10]	N
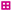	Xgrid	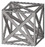	44	15	5	S	S/B	[59]	Y

^1^ Number of struts. ^2^ Number of nodes. ^3^ Maxwell’s number. ^4^ Stretching (S, blue color) or Bending (B, orange color) based on Maxwell’s number. ^5^ Stretching (S, blue color) or Bending (B, orange color) based on the literature references in the next column. (S/B) indicates a mix of Stretching and Bending modes (red color). ^6^ Strut(s) in loading direction? Yes (Y, green color) or No (N, yellow color).

## Data Availability

Data are contained within the article.
